# Genome-Wide Survey of Flavonoid Biosynthesis Genes and Gene Expression Analysis between Black- and Yellow-Seeded *Brassica napus*

**DOI:** 10.3389/fpls.2016.01755

**Published:** 2016-12-06

**Authors:** Cunmin Qu, Huiyan Zhao, Fuyou Fu, Zhen Wang, Kai Zhang, Yan Zhou, Xin Wang, Rui Wang, Xinfu Xu, Zhanglin Tang, Kun Lu, Jia-Na Li

**Affiliations:** ^1^Chongqing Engineering Research Center for Rapeseed, College of Agronomy and Biotechnology, Southwest UniversityChongqing, China; ^2^Engineering Research Center of South Upland Agriculture of Ministry of Education, Southwest UniversityChongqing, China; ^3^Food and Bioproduct Science, University of SaskatchewanSaskatoon, SK, Canada; ^4^Department of Botany and Plant Pathology, Purdue UniversityWest Lafayette, IN, USA

**Keywords:** *Brassica napus* L., Brassicaceae species, expression profile, flavonoid biosynthesis pathway, phylogenetic analysis, seed coat color

## Abstract

Flavonoids, the compounds that impart color to fruits, flowers, and seeds, are the most widespread secondary metabolites in plants. However, a systematic analysis of these loci has not been performed in Brassicaceae. In this study, we isolated 649 nucleotide sequences related to flavonoid biosynthesis, i.e., the *Transparent Testa* (*TT*) genes, and their associated amino acid sequences in 17 Brassicaceae species, grouped into *Arabidopsis* or Brassicaceae subgroups. Moreover, 36 copies of 21 genes of the flavonoid biosynthesis pathway were identified in *Arabidopsis thaliana*, 53 were identified in *Brassica rapa*, 50 in *Brassica oleracea*, and 95 in *B. napus*, followed the genomic distribution, collinearity analysis and genes triplication of them among Brassicaceae species. The results showed that the extensive gene loss, whole genome triplication, and diploidization that occurred after divergence from the common ancestor. Using qRT-PCR methods, we analyzed the expression of 18 flavonoid biosynthesis genes in 6 yellow- and black-seeded *B. napus* inbred lines with different genetic background, found that 12 of which were preferentially expressed during seed development, whereas the remaining genes were expressed in all *B. napus* tissues examined. Moreover, 14 of these genes showed significant differences in expression level during seed development, and all but four of these (i.e., *BnTT5, BnTT7, BnTT10*, and *BnTTG1)* had similar expression patterns among the yellow- and black-seeded *B. napus*. Results showed that the structural genes (*BnTT3, BnTT18*, and *BnBAN*), regulatory genes (*BnTTG2* and *BnTT16*) and three encoding transfer proteins (*BnTT12, BnTT19*, and *BnAHA10*) might play an crucial roles in the formation of different seed coat colors in *B. napus*. These data will be helpful for illustrating the molecular mechanisms of flavonoid biosynthesis in Brassicaceae species.

## Introduction

Rapeseed (*Brassica napus*) is the most important source of edible vegetable oil and protein-rich meal in the world diet, and the seeds, which store protein and oil, are the major harvested plant organ (Nesi et al., 2008). However, the quality of rapeseed oil and meal is greatly affected by the pigments and polyphenols derived from flavonoid biosynthesis that remain after oil extraction. Rapeseed use is limited by the concentration of anti-nutritional factors, including phenolic compounds, lignin, tannins, and proanthocyanidins, it contains. Previous research showed that yellow-seeded *B. napus* has a thinner seed coat, less pigmentation, and higher protein and oil contents than does black-seeded *B. napus* in the same background, rendering it a more nutritional feed for livestock (Chen and Heneen, 1992; Tang et al., 1997; Meng et al., 1998). Thus, selecting lines with a stable yellow-seed trait is one of the most important breeding aims for *B. napus*.

In plant kingdom, flavonoid biosynthesis pathway play important roles in the coloration of fruits, flowers, and seeds, and numerous evidences had showed that *TT*-type genes and their homologs are crucial for the accumulation of flavonoids and their derivatives (Nesi et al., 2001; Winkel-Shirley, 2002; Xie et al., 2003; Baudry et al., 2004; Hoffmann et al., 2006; Lepiniec et al., 2006; Kasai et al., 2007). In the model plant *Arabidopsis thaliana*, the formation of transparent and colorless testa (seed coat) were associated with *tt* loci that are disrupted the flavonoid synthesis pathway in the loss-of-function mutations (Wan et al., 2002; Winkel-Shirley, 2002; Baudry et al., 2004; Lepiniec et al., 2006). To date, 17 genes involved in this pathway have been cloned and functionally characterized, including eight structural genes (i.e., *TT3, TT4, TT5, TT6, TT7, FLS1, LDOX*, and *BAN*; Albert et al., 1997; Devic et al., 1999; Xie et al., 2003; Routaboul et al., 2006; Chiu et al., 2010), six regulatory genes (*TT1, TT2, TT8, TTG1, TTG2*, and *TT16*; Nesi et al., 2000, 2001; Baudry et al., 2006; Routaboul et al., 2006), and three encoding transfer proteins (*TT12, TT19*, and *AHA10*; Debeaujon et al., 2001; Baxter et al., 2005), which were also classified as Early Biosynthetic Genes (*CHS, CHI*, and *F3H* etc.) and Late Biosynthetic Genes (*BAN, DFR*, and *TTG1*, etc.) (Nesi et al., 2000, 2001; Winkel-Shirley, 2001; Lepiniec et al., 2006). Homologs of some of these genes, named *TT*–type genes, have also been identified and shown to be involved in the flavonoid biosynthetic pathway. These genes are thus candidate genes for the molecular basis of seed color manifestation (Supplementary Table S1). *TTG1* in *Brassica rapa* has the same gene function as its orthologs in *A. thaliana*, i.e., it influences root hairiness and the color of the seed coat (Zhang et al., 2009). *BrTT8* was recently shown to regulate the accumulation of proanthocyanidins (PAs) in the seed coat and to regulate the expression of the late biosynthetic genes (LBGs) of the flavonoid pathway in *B*. *rapa*, and an analysis in the “sarson” line of *B*. *rapa* showed that the yellow-seeded trait was caused by loss of *BrTT8* function (Li X. et al., 2012). In addition, *BjuA*.*TT8* and *BjuB*.*TT8* co-segregated perfectly with the seed coat color phenotype in allotetraploid *Brassica juncea* (Padmaja et al., 2014). However, the inheritance of seed coat color is complex in *B*. *napus*. In previous studies, a stable major quantitative trait locus (QTL) for seed coat color of *B*. *napus* was detected in different generations and environments, and *TT10* was considered as a candidate gene involved in seed coat color, based on microsynteny of this QTL with *Arabidopsis* genome sequences (Fu et al., 2007). The following findings showed that *BnTT10* functions in proanthocyanidin polymerization and lignin biosynthesis, as well as seed coat pigmentation in *B*. *napus* (Zhang et al., 2013). Additionally, Chai et al. (2009) found that *TT12* was also a candidate gene for seed coat color in *B*. *napus*. Moreover, several key loci isolated from *B*. *napus* by our group, such as *F3'H, PAL1, TTG1*, and *TT2*, showed no or limited down-regulation in the yellow-seeded lines (Wei et al., 2007; Xu et al., 2007; Ni et al., 2008; Lu et al., 2009). Based on a marker closely linked with a major QTL for seed fiber and color in *B*. *napus*, Stein et al. proposed that the *transparent testa* gene *AHA10* has a strong effect on both seed color and lignin content (Stein et al., 2013). Undoubtedly, the inheritance of seed color in *B*. *napus* is also sensitive to environmental influences, such as lighting, temperature, maturity, and harvest time (Chen and Heneen, 1992; Deynze et al., 1995). Therefore, the molecular mechanism underlying the yellow seed coat trait is unclear in *Brassica* species.

The family Brassicaceae is well known for its large variation in chromosome numbers, common occurrence of polyploids and many reports of interspecific gene flow (Marhold and Lihová, 2006). Moreover, Brassicaceae plants arose form a common ancestor, of which *B*. *napus* (AACC, genome size ~849.7 Mb) was allotetraploid species formed ~7500 years ago by hybridization between *B. rapa* (AA, genome size ~312 Mb) and *Brassica oleracea* (CC, genome size ~540 Mb), followed by genome duplications and mergers during the evolutionary process (Chalhoub et al., 2014). Therefore, *Brassica* is an ideal model to increase knowledge of polyploid evolution (Parkin et al., 2005; Albertin et al., 2006), which is usually assumed that the physiology and developmental biology of *TT* genes in *A. thaliana* are highly similar to those of other Brassicaceae plants. Although dozens of genes involved in the flavonoid biosynthesis pathway of *A. thaliana* were identified based on *tt* mutations (Holton and Cornish, 1995; Devic et al., 1999; Wan et al., 2002; Xie et al., 2003; Baudry et al., 2006; Lepiniec et al., 2006; Routaboul et al., 2006; Saito et al., 2013), only some of these have been characterized in other Brassicaceae plants, and to date no comprehensive study of these genes has been reported. We previously conducted a systematic study of the expression profiles of related genes in *B. napus* seeds at different stages of development (Qu et al., 2013).

**Figure 1:**
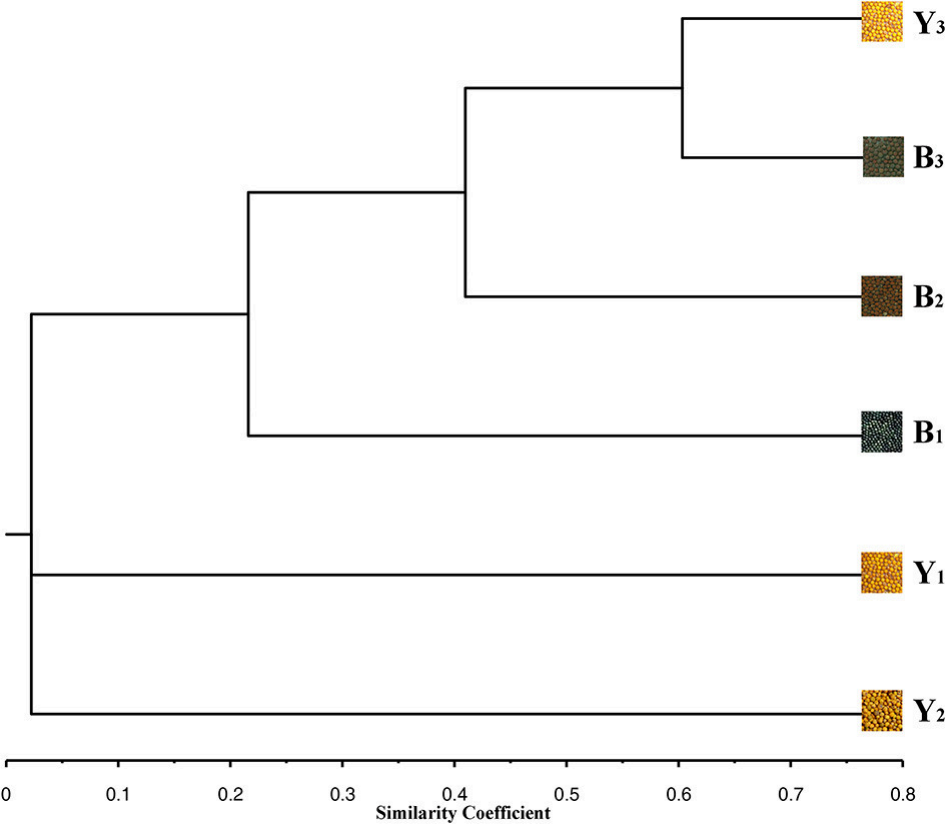
**Phenogram showing Jaccard's genetic similarity coefficients for 6 inbred lines revealed by UPGMA clustering based on genetic fingerprints calculated from 2300 SNP markers**. The phenotypic characteristics of 6 inbred lines were represented by seeds.

In this study, we identified 21 genes involved in the flavonoid biosynthesis pathway in 17 sequenced Brassicaceae species. We systematically analyzed the phylogenetic relationships and triplication events of these genes among the Brassicaceae plants. Additionally, the inheritance of flavonoid biosynthesis pathway in *B. napus* is quite complicated, and the regulatory mechanisms underlying the biosynthesis of the relevant genes were not well understood. Hence, using quantitative real-time PCR (qRT-PCR) analysis, we identified significant differences (Student's *t*-test, *P* < 0.05 or 0.01) in the expression patterns of 18 genes associated with the flavonoid biosynthesis pathway in the stems, leaves, buds, flowers, siliques, and pericarps, and at five different stages of seed development (10, 20, 30, 40, and 50 DAP) in six inbred rapeseed lines, which were used to represent typical yellow- and black-seeded genotypes of *B. napus*, have different genetic backgrounds (Figure 1, Table 1). These results provide useful information for identifying key genes or regulatory nodes that control yellow seed coat formation, and provide insight into the inheritance of qualitative differences between the yellow- and black-seeded *B*. *napus*.

**Table 1:** **List of *B*. *napus* genotypes used in this study**.

**No**.	**Genetic background**	**Yellow seed degree**	**Location**
B_1_	ZY821	22.23	Chongqing, China
Y_1_	GH06	126.43	Chongqing, China
B_2_	ZS9	63.00	Chongqing, China
Y_2_	05E258-1	121.46	Chongqing, China
B_3_	ZS9/06E241(BC_2_F_6_)	54.21	Chongqing, China
Y_3_	ZS9/06E241(BC_2_F_6_)	131.68	Chongqing, China

**Table 2:** **Characterization of genes involved in the phenylpropane-flavonoid biosynthesis pathway of Brassicaceae species**.

**Gene name**	**Length (aa)**	**No. of Brassicaceae Species**	***A. thaliana***	***B. rapa***	***B. oleracea***	***B. napus***	**Total No**.
*PAL1*	263–728	18	1	2	2	4	28
*PAL2*	377–725	16	1	3	4	5	31
*PAL3*	107–698	6	1	2	2	2	10
*PAL4*	344–709	16	1	1	1	2	22
*C4H*	468–1197	17	1	5	5	10	43
*TT1*	287–323	17	1	1	1	2	20
*TT2*	156–265	17	1	1	1	2	20
*TT3*	244–387	18	1	1	1	3	24
*TT4*	393–734	15	1	5	3	9	39
*TT5*	197–254	18	1	3	3	4	30
*TT6*	274–548	16	1	4	3	6	34
*TT7*	364–687	17	1	1	1	2	21
*TT8*	497–613	17	1	1	1	2	23
*TT10*	489–1676	17	1	2	1	3	22
*TT12*	507–560	17	1	1	1	2	22
*TT15*	473–864	17	1	1	1	2	24
*TT16*	183–318	17	1	3	3	6	33
*TT18*	351–416	18	1	2	2	4	28
*TT19*	213–439	17	1	2	2	5	27
*TTG1*	120–521	17	1	2	1	3	24
*TTG2*	114–431	17	1	2	3	4	31
*AHA10*	918–985	16	1	1	1	2	20
*BAN*	216–1041	17	1	2	2	4	27
*UGT2a*	422–566	12	1	1	2	2	14
*UGT2b*	84–562	17	1	4	3	5	35

## Materials and methods

### Plant materials

Plant materials were collected from yellow-seeded and black-seeded *B*. *napus* in different genetic backgrounds (Table 1, Figure 1). The yellow seed degree are calculated using the published reports (Li et al., 2012). The B_1_ and B_2_ were inbred lines ZY821 and ZS11, and Y_1_ and Y_2_ were GH06 and 05E258-1, which were used to represent typical black-seeded and yellow-seeded genetic types of *B*. *napus*, respectively. B_3_ and Y_3_ were represent two near isogenic lines of black- and yellow-seeded *B*. *napus*, selected from the successive backcross of the sixth generation with parent ZS11 and recurrent parent 06E241 (Qu et al., 2015). The plants were grown under normal field conditions at Chongqing Rapeseed Technology Research Center (CRTRC) in 2014. Field management essentially followed normal agronomic procedures. Various tissue organs, including stems (St), leaves (Le), flowers (Fl), buds (Bu), silique pericarps (SP), and seeds harvested at 10, 20, 30, 40, and 50 days after pollination (DAP) were sampled and stored at −80°C until used.

### Identification of flavonoid biosynthesis pathway gene members in brassicaceae species

The full genome sequences of Brassicaceae were downloaded from multiple databases, including the BRAD database (http://brassicadb.org/brad/ftpTrans.php; Cheng et al., 2011), the Phytozome database (http://phytozome.jgi.doe.gov/pz/portal.html#!search; Goodstein et al., 2012), PGDD (PLANT GENOME DUPLICATION DATABASE; http://chibba.agtec.uga.edu/duplication/index/files; Lee et al., 2013), the *Raphanus sativus* Genome DataBase (http://radish.kazusa.or.jp/; Kitashiba et al., 2014), and the *B. napus* database (http://www.genoscope.cns.fr/brassicanapus/; Chalhoub et al., 2014). From amongst the species with full genome sequences, the following 17 species were selected: *Aethionema arabicum* (*Aa*), *Arabidopsis halleri* (*Ah*), *Arabidopsis lyrata* (*Al*), *A. thaliana* (*At*), *Brassica napus L.* (*Bn*), *B. oleracea* (*Bo*), *B. rapa* (*Br*), *Boechera stricta* (*Bs*), *Capsella grandiflora* (*Cg*), *Capsella rubella* (*Cr*), *Camelina sativa* (*Cs*), *Leavenworthia alabamica* (*La*), *Raphanus sativus L.* (*Rs*), *Sisymbrium irio* (*Si*), *Schrenkiella parvula* (*Sp*), *Thellungiella halophile* (*Th*), and *Thellungiella salsuginea* (*Ts*). All coding sequences (CDSs) and amino acid sequences were stored in a local Brassicaceae database using Geneious Pro 4.8.5 software (http://www.geneious.com/; Biomatters, Auckland, New Zealand). To identify the flavonoid biosynthesis pathway genes and their homologous genes, all amino acid sequences of flavonoid biosynthesis pathway genes in *A. thaliana* retrieved from TAIR 10 (http://www.arabidopsis.org/; Lamesch et al., 2012) were used as queries to search against the Brassicaceae protein models with HMMER3 (version 3.1b2 with Pfam HMM library Pfam 28.0; Finn et al., 2011). To identify the flavonoid biosynthesis genes, unique protein sequences of these genes from the *A. thaliana* genome were used as query using BLASTP program (Altschul et al., 1997) in the local Brassicaceae database developed in this research. All taxa were named using two-letter acronyms and gene type was used as the species gene name. Briefly, the first uppercase letter represents the genus, the second the species, and the following the gene name. A number at the end indicates the copy number. For example, *A. thaliana* phenylalanine ammonia lyase 1, which has only one copy in *A. thaliana*, is indicated by *AtPAL1*.

### Mapping of flavonoid biosynthesis pathway genes among brassicaceae species

To assign the location of flavonoid biosynthesis pathway genes in the Brassicaceae species genomes, the GFF genome files were downloaded from the aforementioned databases. Then, MapChart 2.0 was used to draw graphic representations of their corresponding physical position on pseudo-molecular chromosomes of *Brassica* crops.

### Phylogenetic analysis

Based on previously described methods, all sequence alignments for each flavonoid biosynthesis pathway gene superfamily were performed using ClustalW2 software (Larkin et al., 2007), and phylogenetic analysis was carried out using Molecular Evolutionary Genetics Analysis (MEGA) 6.0 (Tamura et al., 2013) with a maximum likelihood (ML). In the ML method, phylogenetic trees were constructed using the JTT+I+G substitution model in PhyML version 3.0.1. To ensure the accuracy of the phylogenetic tree, each tree was subjected to bootstrap analysis with 1000 replicates (Guindon et al., 2010). Finally, all the phylogenetic trees were visualized using FigTree v1.4.2 (http://tree.bio.ed.ac.uk/software/figtree/).

### Expression pattern analysis of *TT*-type genes in *B. napus*

To characterize differences in expression of the 18 genes associated with the flavonoid biosynthesis pathway between the yellow- and black-seeded varieties, we designed the primers in consensus region based on alignment the gene sequence (Table 3). Then the total RNA was extracted from various tissues using the RNAprep Pure Plant Kit (*TIANGEN BIOTECH*, Beijing, China) according to manufacturer's instructions. Then, 1 μg RNA sample was reverse transcribed with the Oligo dT-Adaptor Primer using the RNA PCR Kit (AMV) Ver. 3.0 (TaKaRa, http://www.takara.com.cn). To monitor sample uniformity of initial RNA input and RT efficiency, *Brassica napus 26S rRNA* was used as the internal control according to the previously described method (Qu et al., 2013).

**Table 3:** **Primers of the flavonoid biosynthesis pathway genes and housekeeping gene used for qRT-PCR**.

**Target gene**	**Forward sequence (5′–3′)**	**Reverse sequence (5′–3′)**	**GenBank**	**AGI number**
*BnTT4*	GACTACTACTTCCGCATCACCAACAG	GCCTAGCTTAGGGACTTCAACAACC	AF076335	AT5G13930
*BnTT5*	CTTCCTCGGTGGCGCAGGTG	ACACAGTTCTCCGTTACTTTCTCTGA	EU402417	AT3G55120
*BnTT6*	TGGGTGAAAGTGACGGAGGAGT	TGGTTCCAGGGTCAGTGTGACG	DQ513329	AT3G51240
*BnTT7*	GCCATAGCCCGTGACCCGGA	GCTTCTCCGGCGTAACTCCTCC	DQ324379	AT5G07990
*BnTT3*	AGACCGTGTGCGTAACCGGC	AGGATCGCGAACAGTGGCACG	DQ767950	AT5G42800
*BnTT18*	GGCTTAGAGCCTGACCGTCTAGAGAA	TGAGCTTCCACGCCAAGTGCT	GQ120562	AT4G22880
*BnBAN*	GGACTTGTGATGACCGAAGAAAACTG	ATGTAGCGACCAGAAGCTGTTTCTTT	FJ938339	AT1G61720
*BnTT12*	GCTCCACAGAGACATACGAGCCG	ACGGTGACGAAGCTGAGCATGTA	EU818785	AT3G59030
*BnTT19*	ACATCTTCTTCGTCAGCCATTTGGTCA	GGTCCACGATGGCTCGGTGC	AB117793	AT5G17220
*BnTT10*	GCGACTGTGCCAAGAAACGGT	CCCCACGTGAGATGTCTATCAAAGTG	HM805059	AT5G48100
*BnAHA10*	ACCCATTGCCATGCCCACTGT	GCTCGGCCTGCAAGCAACAA	NM_101587	AT1G17260
*BnTT2*	AGCTGGTCTCAAGAGGTGTGGCA	AGCCTCCCAGCTATCAACGACC	DQ778647	AT5G35550
*BnTT8*	GGCTGAAGAGGCTGCGTCGG	GTGCTGTGCAAGCCCTCGCT	EU192027	AT4G09820
*BnTTG1*	TCCTCCGGCGACTTCCTCCG	GCTGCGTCTCCACCACGGAC	EF175930	AT5G24520
*BnTT16*	TGCTCACATCGGTCTCATCGTCT	GCTCGTGTGGAGGAATGGAGGC	EU192028	AT5G23260
*BnTTG2*	AAACCTAAAGCAAAGCTTGTCTCCCA	ACTTCCTTTGACTTGCTTCTGTCCGT	FJ012168	AT2G37260
*BnTT1*	TCGCTACAACAATCTTCAGATGCACA	TCCTGCACCCTTCAACGCAGC	AF190298	AT1G34790
*BnTT15*	ACAAAATGACGGGACAGTGGAAGTT	GGCTGCACATCGCCTCGAGTT	BT005834	AT1G43620
*BnACTIN7*	TGGGTTTGCTGGTGACGAT	TGCCTAGGACGACCAACAATACT	EV116054	AT5g09810
*BnUBC21*	CCTCTGCAGCCTCCTCAAGT	CATATCTCCCCTGTCTTGAAATGC	EV086936	AT5g25760

Primers for amplifying partial sequences of flavonoid biosynthesis genes were designed from conserved nucleotide regions identified by multiple alignments of sequences.

Real-time PCR was performed using SYBR® Premix Ex Taq™ II (Perfect Real Time) (TaKaRa, China) in a 20 μl volume that included 10 μl of SYBR® Premix Ex Taq™ II, 2 μl (100 ng) of template cDNA, and 0.4 μM of each PCR primer. All primer sequences used for the qRT-PCR are listed in Table 3, designed according to the methods described in our previous research (Qu et al., 2013). Then the specific primers used in this study and cycling conditions were 95°C for 2 min, followed by 40 cycles at 95°C for 10 s (denaturation) and 60°C for 20 s (annealing and extension). The melting curves of each PCR application were obtained using the following cycling conditions: 95°C for 10 s followed by a constant increase in temperature between 65 and 95°C at an increment of 0.5°C/cycle, and samples were run on the Bio-Rad CFX96 Real Time System (USA). The relative expression of the target genes was analyzed using the 2^−Δ*ΔCt*^ method with *BnACTIN7* (EV116054) and *BnUBC21* (EV086936) as internal controls (Wu et al., 2010). Three biological replicates for each sample were used for real-time PCR analysis and three technical replicates were analyzed for each biological replicate. Then the values represent the average ± SD of three biological replicates with three technical replicates of each tissue and organ. Relative gene expression levels were normalized according to the expression values in black-seeded at 10 DAP.

## Results

### Characterization of flavonoid biosynthesis pathway gene members in brassicaceae species

To identify members of the Brassicaceae gene subfamily that are involved in the flavonoid biosynthesis pathway, the whole genome sequences of 17 species were downloaded from multiple public databases (Materials and Methods). In total, 649 nucleotide sequences of 21 genes and their associated amino acid sequences were respectively were identified using the HMM profile (Finn et al., 2011). In addition, we used the nucleotide and encoded amino acid sequences of four genes from *B. juncea, Bj_PAL1* (ACX31148.1), *Bj_TT3* (ADB45307.1), *Bj_TT5* (ADB45305.1), and *Bj_TT18* (ACH58397.1), that we identified and aligned previously (Qu et al., 2013). The copy numbers of each gene varied from 20 to 91, and the genes were widely distributed in the 17 Brassicaceae species examined. The encoded amino acid sequences ranged from 84 aa (*Bn_UGT2d*) to 1676 aa (*Sp_TT10*; Supplementary Table S2). In addition, the number of gene families also varied by species; for example, only 10 copies of *PAL3* were found in 6 species, but 43 *C4H* gene copies occurred in 17 species with as many as 10 orthologs (*BnC4H*) in *B. napus* (Supplementary Table S2). However, *TT4, TT6*, and *AHA10* were not identified in all Brassicaceae species. For example, *TT4* was not found in *A. halleri* and *C. rubella, TT6* was absent from *A. halleri*, and *AHA10* was not present in *A. lyrata* (Supplementary Table S2). We used the sequences of flavonoid biosynthesis genes from *A. thaliana* as query to search genome databases, and identified highly conserved sequences, including *LESS ADHESIVE POLLEN*5/6 (*LAP5/6*) and *TT4* in *A. halleri* and *C. rubella, DMR6-LIKE OXYGENASE*1/2 (*DLO1/2*) and *TT6* in *A. halleri*, and members of the Autoinhibited H(+)-ATPase (AHA) superfamily, which might be homologs of *TT4, TT6*, and *AHA10* and function in flavonoid biosynthesis in *A. lyrata*. Here, 36 copies of 21 genes of the flavonoid biosynthesis pathway were identified in *A. thaliana*, but 53 were identified in *B. rapa*, 50 in *B. oleracea*, and 95 in *B. napus*, in accordance with the fact that sequences present as a single copy in the *A. thaliana* were present in 2–8 copies in *B. napus* (Cavell et al., 1998), and with the observation that excessive gene loss is typical after polyploidization in eukaryotes (Sankoff et al., 2010; Wang et al., 2011).

### Genomic distribution on chromosomes of brassicaceae species

**Figure 2:**
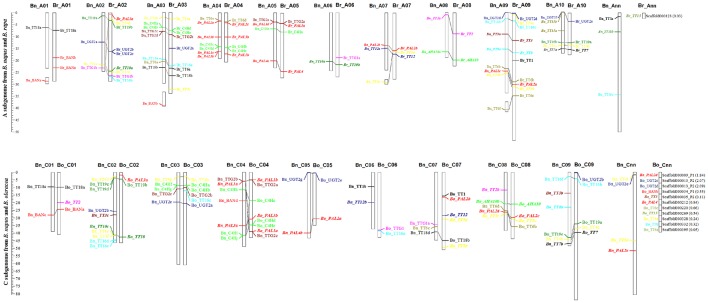
**Genomic distribution of the flavonoid biosynthesis pathway genes on *B. rapa, B. oleracea*, and *B. napus* chromosomes**. The dissociation fraction of chromosomes indicates the normal single-copy locus mapped to the random chromosome; Ann and Cnn are the indeterminate Chromosome A and C. Numbers in parentheses are the physical distance of the scaffold for each gene. The collinearity of homologous genes were indicated by the ligatures among *Brassica* species. The homologous genes of flavonoid biosynthesis pathway were indicated by the same colors of the exact same fonts among different chromosomes of *Brassica* species.

All of the flavonoid biosynthesis pathway genes in 17 Brassicaceae species were mapped onto pseudo-molecules or chromosomes using GFF files of their nucleotide sequences (Supplementary Table S3). *Brassica* crops are the ideal model for studying genome evolution (Wang et al., 2011). To intuitively assign the physical position to the chromosomes of *B. rapa, B. oleracea*, and *B. napus*, all gene members of flavonoid biosynthesis pathway were mapped to their chromosomes [52 (98.1%) gene copies in *B. rapa*, 39 (78.0%) gene copies in *B. oleracea*, and 84 (88.4%) gene copies in *B. napus*] and 22 (1 in *B. rapa*, 11 in *B. oleracea*, and 11 in *B. napus*) were distributed on the unanchored scaffolds (Figure 2, Supplementary Table S3), which showed strong collinearity between the A subgenomes from *B. rapa* and *B. napus* and the C subgenomes from *B. oleracea* and *B. napus* (Figure 2). This result indicates that the gene copies are distributed in orthologous blocks in each genome, and that substantial genome reshuffling had occurred. For example, copies of *TT10* and *TT16* were not identified on *B. napus* chromosome A02, and the differential gene copies located in orthologous blocks on chromosome C06 differed greatly between *B. oleracea* and *B. napus* (Figure 2). These findings are consistent with the fact that Brassicaceae genomes underwent Brassicaceae-lineage-specific whole genome triplication, followed by diploidization after divergence from their common ancestor (Lysak et al., 2005; Town et al., 2006; Mun et al., 2009; Wang et al., 2011; Cheng et al., 2013).

### Phylogenetic analysis and the triplication of the flavonoid biosynthesis pathway gene superfamily

Despite different flavonoid biosynthesis pathway genes having similar functions in Brassicaceae species, the phylogenetic relationships of members of each superfamily have not been comprehensively studied. To gain insight into the functional roles of flavonoid biosynthesis pathway genes that may have arisen during evolution, we performed a phylogenetic analysis using the putative amino acid sequence based on each member of the flavonoid biosynthesis pathway superfamily in Brassicaceae species.

Initially, we conducted a BLASTP analysis against the local database, which included 17 Brassicaceae species, followed by HMM searching (Finn et al., 2011). We identified 649 sequences of 21 flavonoid biosynthesis pathway gene family members, and found that the number of gene copies varied from 1 to 10 in each family (Supplementary Table S2), which were mapped onto pseudo-molecules or chromosomes using GFF files of their nucleotide sequences (Supplementary Table S3). To intuitively assign the physical position to the chromosomes of *B. rapa, B. oleracea*, and *B. napus*, all gene members of flavonoid biosynthesis pathway were mapped to their chromosomes [52 (98.1%) gene copies in *B. rapa*, 39 (78.0%) gene copies in *B. oleracea*, and 84 (88.4%) gene copies in *B. napus*] and 22 (1 in *B. rapa*, 11 in *B. oleracea*, and 11 in *B. napus*) were distributed on the unanchored scaffolds (Figure 2, Supplementary Table S3), which showed strong collinearity between the A subgenomes from *B. rapa* and *B. napus* and the C subgenomes from *B. oleracea* and *B. napus* (Figure 2). This result indicates that the gene copies are distributed in orthologous blocks in each genome, and that substantial genome reshuffling had occurred. For example, copies of *TT10* and *TT16* were not identified on *B. napus* chromosome A02, and the differential gene copies located in orthologous blocks on chromosome C06 differed greatly between *B. oleracea* and *B. napus* (Figure 2). These findings are consistent with the fact that Brassicaceae genomes underwent Brassicaceae-lineage-specific whole genome triplication, followed by diploidization after divergence from their common ancestor (Lysak et al., 2005; Town et al., 2006; Mun et al., 2009; Wang et al., 2011; Cheng et al., 2013).

**Figure 3:**
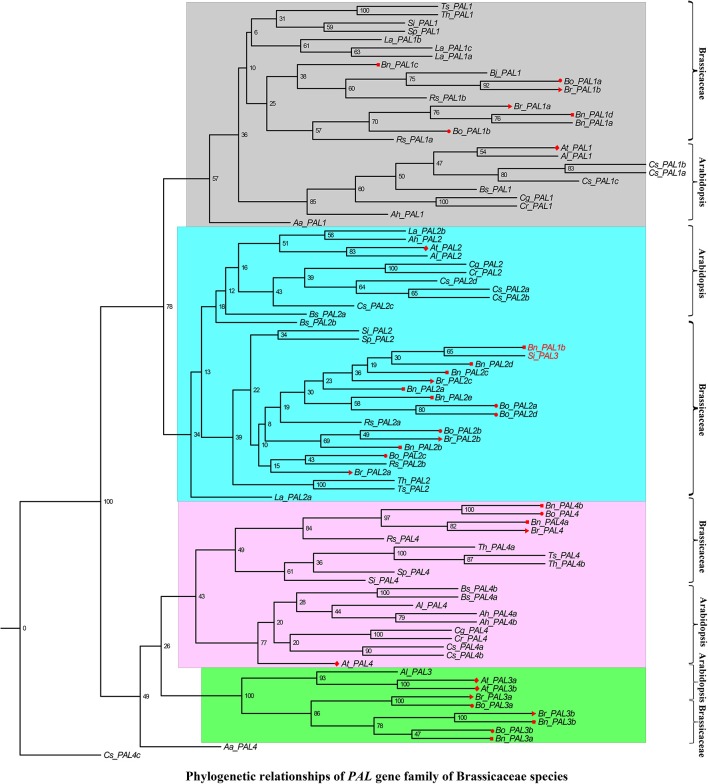
**Phylogenetic relationships of *PAL* gene family of Brassicaceae species**. *PAL1, PAL2, PAL3*, and *PAL4* were indicated by gray, light blue, purple and green color. The *Bn_PAL1b* and *Si_PAL3* were denoted by red font that maybe the synonymous of *PAL2*. The Red color diamond, triangle, circle, and rectangle were denoted the gene copies in *A. thaliana, B. rapa, B. oleracea*, and *B. napus*, respectively. Scale bar (the numbers) indicates the estimated number of amino acid substitutions per site.

**Figure 4:**
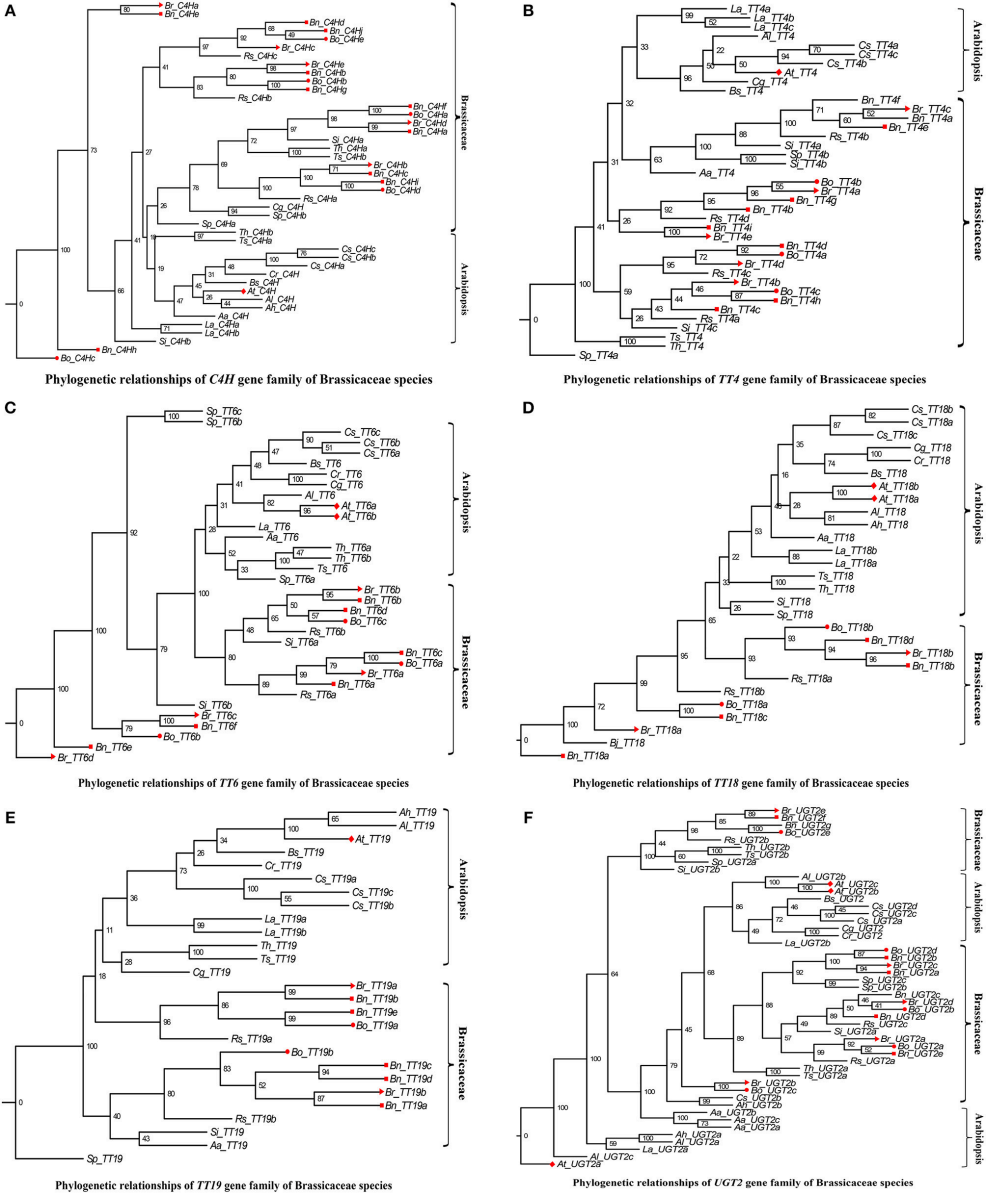
**Phylogenetic relationships of *C4H*, *TT4*, *TT6*, *TT18*, *TT19*, and *UGT2*gene family of Brassicaceae species**. The Red color diamond, triangle, circle, and rectangle were denoted the gene copies in *A. thaliana, B. rapa, B. oleracea*, and *B. napus*, respectively. Scale bar (the numbers) indicates the estimated number of amino acid substitutions per site. **(A–F)** were indicated *C4H, TT4, TT6, TT18, TT19*, and *UGT2* gene family, respectively.

**Figure 5:**
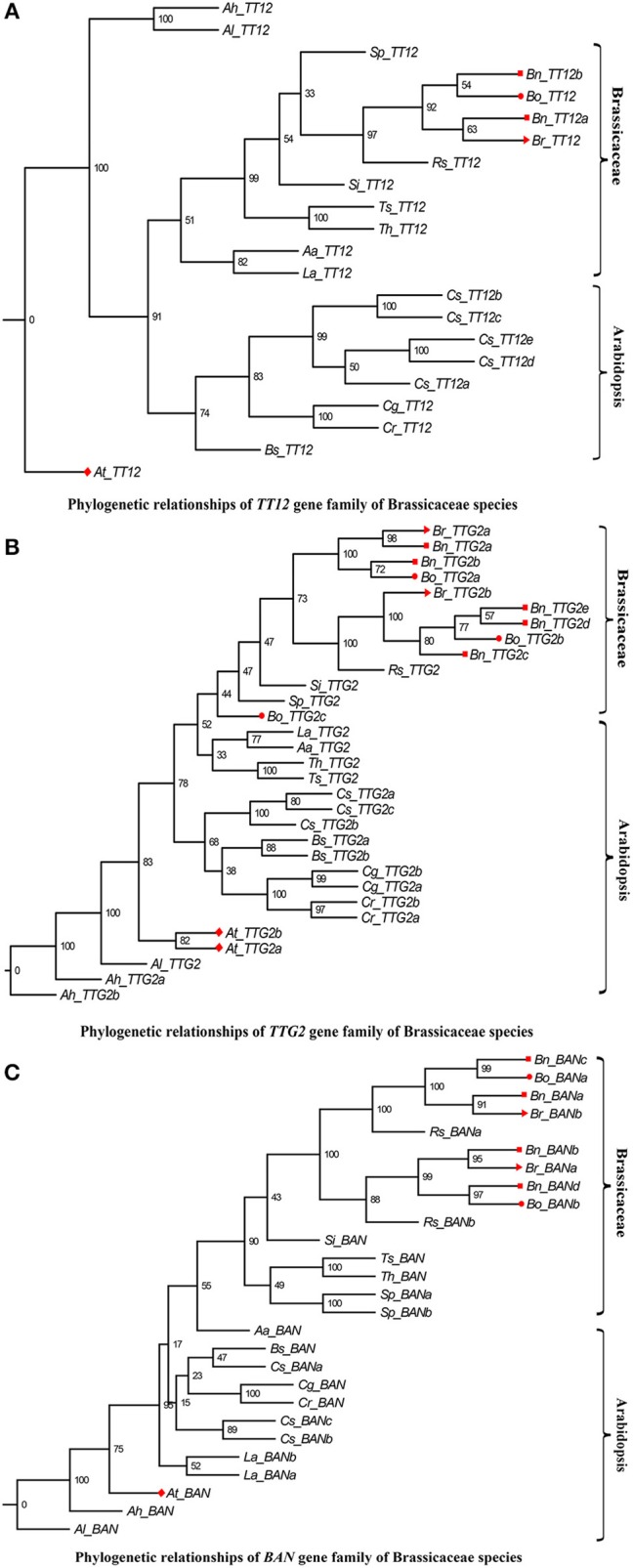
**Phylogenetic relationships of *TT12, TTG2*, and *BAN* gene family of Brassicaceae species**. The Red color diamond, triangle, circle, and rectangle were denoted the gene copies in *A. thaliana, B. rapa, B. oleracea*, and *B. napus*, respectively. Scale bar (the numbers) indicates the estimated number of amino acid substitutions per site. **(A–C)** were indicated *TT12, TTG2*, and *BAN* gene family, respectively.

In addition, we performed a phylogenetic analysis to identify each flavonoid biosynthesis pathway gene type, and constructed their corresponding phylogenetic trees using MEGA 6.0 with the ML method and modified the tree using FigTree v1.4.2. According to the phylogeny generated using the ML method, all representative sequences for each gene member from the Brassicaceae species formed a well-supported clade, which was classified into the *Arabidopsis* or Brassicaceae subgroups, but each gene was assigned to monophyletic clades using other substitution models (Figures 3–5, and Supplementary Figures S1–S10). For example, we identified 4 members of *PAL*, which encodes key enzymes of the phenylpropanoid pathway, that were clearly grouped into four subclades (Figure 3). The gene members, *Bn_PAL1b* and *Si_PAL3* belonged to the same *PAL2* subcategories, and *Aa_PAL4* and *Cs_PAL4c* clustered alone or in the vicinity of the *PAL3* and *PAL4* group (Figure 3; *PAL1*, Gray; *PAL2*, Light blue; *PAL3*, Pink; *PAL4*, Green). We could thus predict the functions of specific flavonoid biosynthesis pathway genes in Brassicaceae species, because genes with similar functions tend to be retained with orthologous genes (Koonin, 2005). In addition, *Brassica* species are an ideal model for systematically studying polyploidy genome evolution. We identified one clade of genes of the flavonoid biosynthesis pathway, and the copies in *B. rapa, B. oleracea*, and *B. napus* (e.g., *C4H, TT4, TT6, TT18, TT19*, and *UGT2)* were divided into different subclades or monophyletic subclades by phylogenetic analysis (Figures 4A–F). However, multiple gene copies of *TT12, TTG2*, and *BAN* were present in the subclades in *B. rapa, B. oleracea*, and *B. napus*, which have high levels of divergence from their common ancestor with *A. thaliana* (Figures 5A–C).

Based on the collinearity of orthologs, we identified syntenic orthologous genes of the flavonoid biosynthesis pathway among the genomes of the Brassicaceae species (Supplementary Table S4). In the genomes of *B. rapa, B. oleracea*, and *B. napus*, the copies of *C4H* were distributed in the LF, MF1, and MF2 subgenomes. Besides, the copies of *C4H* were also identified outside the genome triplication segments in the genomes of *Brassica* crops. However, 10 of 25 gene members had a single copy in the *B. rapa* and *B. oleracea* genomes, but multiple copies in *B. napus*. Moreover, we identified 10 pseudo-copies of five genes in seven species, i.e., 3 of which in *C. rubella* (*C4H, TT10*, and *UGT2*), 2 copies of *TT1* in *L*. *alabamica* and *S*. *irio*, and 5 copies of *TT6* in *A*. *arabicum, B. rapa, B. oleracea*, and *B. napus* (Supplementary Table S4). These findings can be used to systematically study gene retention in the triplicated genomes of Brassicaceae species, as well as to understand the evolutionary history of these orthologous genes among Brassicaceae species.

### Development- and tissue-specific expression of *TT*-type genes in *B. napus*

**Figure 6:**
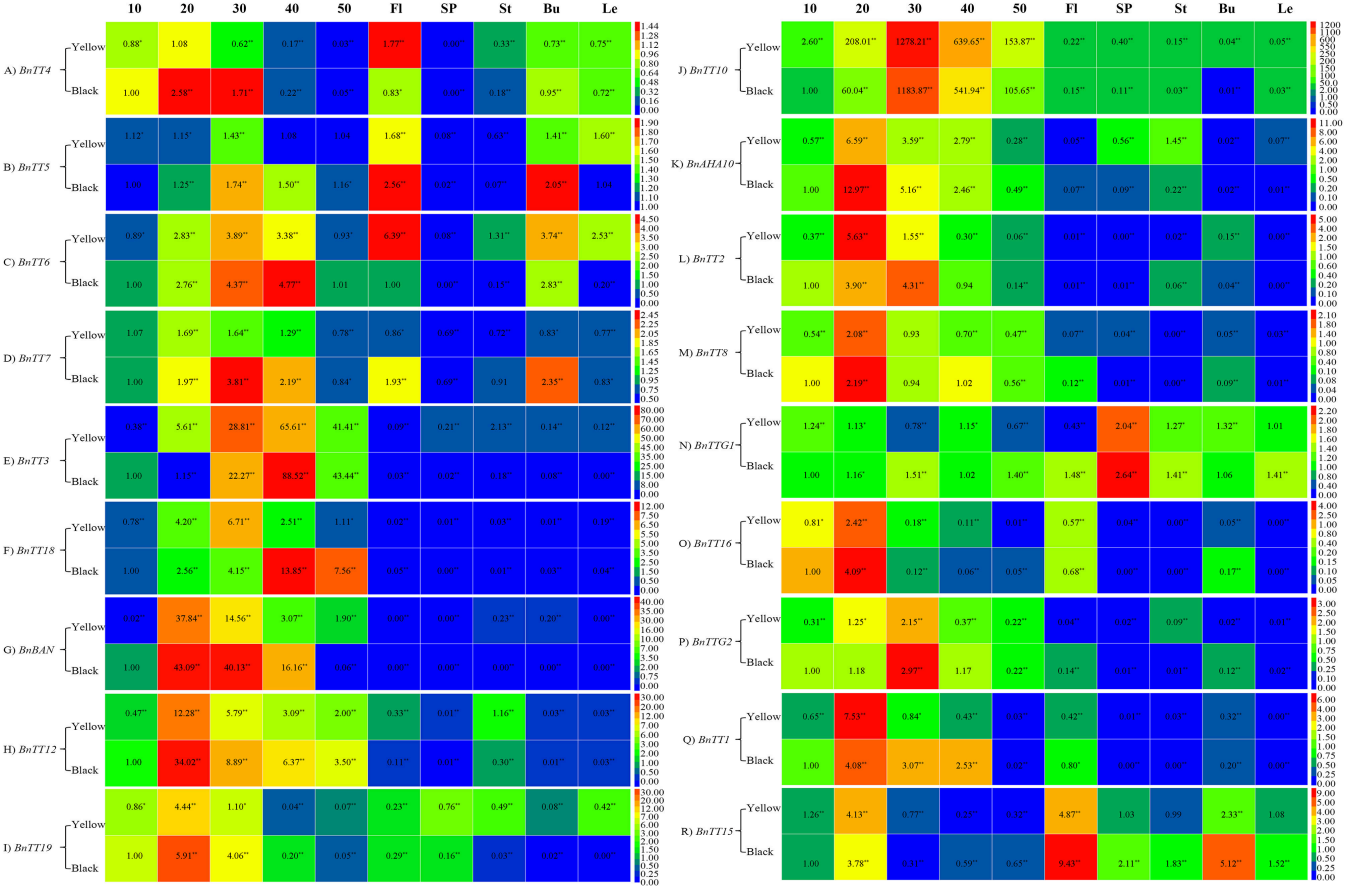
**Comparison expression analysis of genes involved in flavonoid biosynthesis in different tissues and organs between the yellow- and black-seeded *B. napus***. Three biological replicates for each sample were used for real-time PCR analysis and three technical replicates were analyzed for each biological replicate. Values represent the average ± SD of three biological replicates with three technical replicates of each tissue and organ. 10–50, Different stages of seed development; Fl, Flowers; SP, Silique pericarps; St, Stems; Bu, Buds; Le, Leaves. Red indicates high expression and blue indicates low expression. **(A–R)** were indicated the genes of *BnTT4, BnTT5, BnTT6, BnTT7, BnTT3, BnTT18, BnBAN, BnTT12, BnTT19, BnTT10, BnAHA10, BnTT2, BnTT8, BnTTG1, BnTT16, BnTTG2, BnTT1*, and *BnTT15*, respectively.

In this study, we analyzed the expression patterns of 18 *TT*-type genes involved in flavonoid biosynthesis by qRT-PCR in various tissues of black- and yellow-seeded lines of *B*. *napus* with different genetic backgrounds. Except for *BnTT4, BnTT5, BnTT6*, and *BnTT7*, the expression level of 11 structural genes was much higher in developing seeds than in other tissues in *B*. *napus* (Figure 6). The expression level of *BnBAN, BnTT12, BnTT19*, and *BnAHA10* peaked during the early stages of seed development, which were the EBGs (Figures 6G–I,K, Table 4). *BnTT3*, and *BnTT18* were expressed in a similar pattern as *BnBAN, BnTT12, BnTT19*, and *BnAHA10*, but peaked later in development, which were the LBGs (Figures 6E,F,J, Table 4). Thus, these genes may be regulated by the same upstream gene or they may have a synergistic effect on the flavonoid biosynthetic pathway during seed development. In addition, the expression levels of *BnTT5* and *BnTT7* did not differ significantly (Student's *t*-test, *P* > 0.05) among the rapeseed tissues, whereas *BnTT4, BnTT5, BnTT6*, and *BnTT7* expression was higher in the buds and flowers of *B*. *napus* (Figures 6A–D, Table 4). Furthermore, the expression level of five regulatory genes, *BnTT2, BnTT8, BnTT16, BnTTG2*, and *BnTT1*, was much higher in developing *B*. *napus* seeds, and peaked during the early and middle stages of seed development, which may be the EBGs (Figures 6L,M,O,P,Q, Table 4). However, *BnTT15* was expressed at higher levels in flowers than in other organs, indicating that this gene may play an important role in flower formation (Figure 6R).

**Table 4:** **The tissue specificity and expression stages of flavonoid biosynthesis pathway genes in *B*. *napus* and *A. thaliana***.

**Name**	**Tissue specificity**		**Expressed during**	
	***B. napus***	***A. thaliana***	***B. napus***	***A. thaliana***
*TT4*	Except Silique pericarps	Whole plant	EBGs	EBGs
*TT5*	Whole plant	Whole plant	NA	EBGs
*TT6*	Whole plant	Whole plant	LBGs	LBGs
*TT7*	Whole plant	Whole plant	NA	LBGs
*TT3*	Development seeds	Whole plant	EBGs	EBGs
*TT18*	Development seeds	Development seeds	LBGs	LBGs
*BAN*	Development seeds	Development seeds	EBGs	EBGs
*TT12*	Development seeds	Development seeds	EBGs	EBGs
*TT19*	whole plant	Whole Plant	EBGs	EBGs
*TT10*	Development seeds	Development seeds and flarol organs	NA	LBGs
*AHA10*	Development seeds and stem	Development seeds and stem	EBGs	EBGs
*TT2*	Development seeds	Development seeds	EBGs	EBGs
*TT8*	Development seeds	Development seeds	EBGs	EBGs
*TTG1*	whole plant	whole plant	LBGs	LBGs
*TT16*	Development seeds and flowers	Development seeds and flowers	EBGs	EBGs
*TTG2*	Whole plant	Whole plant	LBGs	LBGs
*TT1*	Development seeds and floral organs	Development seeds	EBGs	EBGs
*TT15*	Whole plant	Whole plant	LBGs	LBGs

EBGs and LBGs means the Early Biosynthetic Genes and the Late Biosynthetic Genes, respectively. NA indicates that the expression stage of genes were inconclusive during the developmental seeds of B. napus.

### Differential expression patterns of *TT*-type genes in *B. napus*

**Figure 7:**
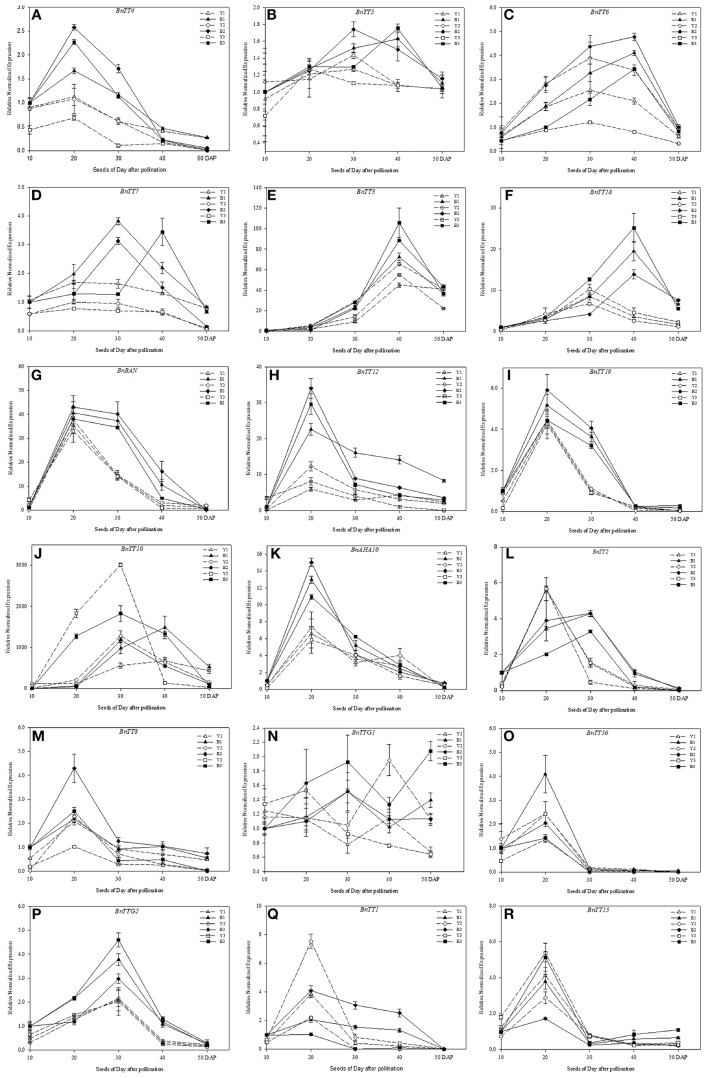
**Expression pattern analysis of genes involved in flavonoid biosynthesis in the developing seeds of different *B*. *napus* lines by qRT-PCR**. The yellow-seeded lines include Y1, Y2, and Y3, and the black-seeded lines include B1, B2, and B3 **(A–R)**. Expression was monitored at five stages of seed development (10, 20, 30, 40, and 50 DAP). Values represent the average ± SD of three biological replicates with three technical replicates of each developmental stage. Error bars denote standard error of the mean (SEM) of three experiments. Relative gene expression levels were normalized according to the expression values in black-seeded *B. napus* at 10 DAP.

In this study, most of the key genes involved in flavonoid biosynthesis were found to exhibit similar expression patterns, and the expression levels of genes varied greatly between the yellow- and black-seeded lines of *B*. *napus* (Figure 7). The structural genes involved in flavonoid biosynthesis, i.e., *BnTT3, BnTT18, BnBAN, BnTT12, BnTT19*, and *BnAHA10*, had similar expression patterns in the developing seeds of different genetic backgrounds, but had significant differences (Student's *t*-test, *P* < 0.01) in expression level between the black- and yellow-seeded lines (Figures 7C,E–I,K, Table 4). *BnTT4, BnBAN, BnTT12, BnTT19*, and *BnAHA10* expression peaked at 20 DAP, which were EBGs (Figures 7A,G–I,K, Table 4), and *BnTT6, BnTT3*, and *BnTT18* expression peaked at 40 DAP, were belong the LBGs (Figures 7E,F, Table 4). These genes were expressed at higher levels in the black-seeded than in the yellow-seeded lines, especially at 20 DAP (Figures 7A,H,K, Table 4), 30 DAP (Figures 7G,I), and 40 DAP (Figures 7B,E,F, Table 4), but the expression patterns of these genes were hardly affected by genetic background. Additionally, *BnTT7, BnTT5*, and *BnTT10* expression peaked at different development stages both in the black- and yellow-seeded lines (Figures 7B,D,J, Table 4, and Supplementary Table S5), indicating that the expression of these genes was affected by the genetic background. As for the expression patterns of structural genes, regulatory genes, such as *BnTT2, BnTT8, BnTT16, BnTTG2, BnTT1*, and *BnTT15*, had similar expression patterns in the black- and yellow-seeded *B*. *napus*, with the genetic background having a minimal effect, and the expression of these genes peaked during the early and middle developmental stages (20 and 30 DAP, EBGs; Figures 7L,M,O–R, Table 4). In addition, the expression patterns and levels of *BnTTG1* showed obvious variations during seed development of different lines of *B. napus*, suggesting that they were significantly affected by genetic background (Figure 7N).

Although the expression patterns of most of the genes involved in flavonoid biosynthesis did not significantly differ (Student's *t*-test, *P* > 0.05) between the developing seeds of the black- and yellow-seeded *B*. *napus*, the expression levels of these genes did differ and peaked at different developmental stages (Figure 7), indicating that these genes can also be classified as Early Biosynthetic Genes and Late Biosynthetic Genes, as in *Arabidopsis* (Lepiniec et al., 2006). The expression of genes that function upstream in the flavonoid biosynthesis pathway peaked before those that functioned later, suggesting that the downstream genes were influenced by the upstream genes. For example, the expression of *BnTT4*, which encodes a protein that catalyzes the first committed step of flavonoid biosynthesis (Albert et al., 1997; Tang et al., 1997), peaked at 20 DAP, which was before the expression of the downstream genes *BnTT5, BnTT6*, and *BnTT7* peaked (Figure 7). All flavonoids and isoflavonoids are derived from the nargingenin chalcone generated by this first enzyme. Moreover, the expression of most of the regulatory genes also peaked at 20 DAP (Figure 7), suggesting that they may be essential for regulating the expression of genes involved in flavonoid biosynthesis during the early stages of seed development. Although, the expression of *BnTTG2* peaked later than did that of *BnTT2* and *BnTT8*, the expression of *BnTT2* was greater in yellow- than in black-seeded *B*. *napus*, and *BnTTG1* expression was also largely affected by the genetic background (Figures 7L–N,P), indicating that the mechanism of *BnTTG2* is different from that of its orthologs in *A. thaliana* (Lepiniec et al., 2006). In addition, the expression of *BnTT6, BnTT18*, and *BnTT2* peaked sooner in the yellow-seeded than in the black-seeded lines (Figures 7C,F,L).

## Discussion

Flavonoids are secondary metabolites that are extensively distributed in the plant kingdom. They not only play an important role in color formation in fruits and flowers, but are also well-known for their positive effect on health, due to their antioxidant and antitumor properties (Winkel-Shirley, 2001, 2002; Lepiniec et al., 2006; Routaboul et al., 2006). Genome-wide analyses of gene superfamilies have been widely performed after the completion of numerous plant genome projects. For instance, numerous genome-wide studies of gene superfamilies have been performed in the diploid species, *B. rapa* and *B. oleracea* (Song et al., 2013; Duan et al., 2014; Li et al., 2014; Diehn et al., 2015; Lu et al., 2015). Several gene superfamilies have also been reported in the allotetraploid species *B. napus* (Sun et al., 2014; Raboanatahiry et al., 2015) However, a comprehensive analysis of the superfamily of genes underlying the flavonoid biosynthesis pathway in *Brassica* has not been reported to date. Here, we identified 649 nucleotide sequences of 21 flavonoid biosynthesis pathway genes and their corresponding amino acid sequences in 17 Brassicaceae species (Table 2, Supplementary Table S2). The gene copy numbers differed between species; for example, 10 copies of *PAL3* were found in 6 species, whereas 43 copies of *C4H* were identified in 17 species (Table 2, Supplementary Table S2). Although we did not detect orthologs for some genes involved in flavonoid biosynthesis (e.g., *TT4, TT6*, and *AHA10*), in accordance with the fact that the excessive gene loss is typical after polyploidy formation in eukaryotes (Sankoff et al., 2010; Wang et al., 2011), we identified orthologs of many of these genes by querying the *Brassica* genome databases with the sequences of *A. thaliana* genes involved in flavonoid biosynthesis. In addition, it has been widely suggested that the genome structures are highly conserved among *Brassica* species (Krishnamurthy et al., 2014; Thamilarasan et al., 2014; Dong et al., 2016). Each of the gene copies was found to be distributed in orthologous blocks by collinearity analysis between the A and C subgenomes (Figure 2). Not all gene members could be accurately annotated on chromosomes and the number of gene copies varied greatly in the orthologous blocks (Figure 2, Supplementary Table S3), indicating that may be associated with Brassicaceae-lineage-specific whole genome triplication, followed by diploidization after divergence from the common ancestor (Lysak et al., 2005; Town et al., 2006; Mun et al., 2009; Wang et al., 2011; Cheng et al., 2013). Although, subgenome sequences present higher levels of conservation in extensive collinear genome blocks among Brassicaceae species, we found that all genes of the flavonoid biosynthesis pathway were phylogenetically classified into two major subcategories (*Arabidopsis* and Brassicaceae species; Figures 3–5, Supplementary Figures S1–S11), consistent with the functional divergence of orthologous gene groups between *Arabidopsis* and Brassicaceae species during evolution. These results revealed that diversification occurred among the flavonoid biosynthesis pathway gene family members, likely indicating that functional divergence of orthologous gene groups occurred between *Arabidopsis* and Brassicaceae species during evolution. These findings provide insight into the functional divergence of these genes among Brassicaceae species. In addition, the identification of conserved genomic blocks will provide useful phylogenetic, polyploidization, and comparative genomics information (Schranz et al., 2006; Cheng et al., 2013). Subgenomes can be classified based on gene density into the following three groups: least fractionated (LF), medium fractionated (MF1), and most fractionated (MF2) (Wang et al., 2011; Cheng et al., 2013). We then performed the triplication of flavonoid genes in the whole gennomes of Brassicaceae species. Furthermore, 10 pseudo-copies of five flavonoid biosynthesis pathway genes were identified, such as *C4H, TT10, UGT2, TT1*, and *TT6* (Supplementary Table S4). These results will provide detailed information for systematic studies of the functions and roles of these genes in flavonoid biosynthesis pathway at the molecular level.

Seed coat color was previously reported to involve a similar mechanism in *Brassica* and *Arabidopsis* species (Marles and Gruber, 2004). Hence, identifying candidate genes by cloning *Brassica TT* genes involved in the flavonoid biosynthetic pathway and conducting comparative studies of these genes is a reasonable approach, and many homologs of these genes have also been identified in *B. napus* (Wei et al., 2007; Xu et al., 2007; Ni et al., 2008; Chai et al., 2009; Lu et al., 2009; Chen et al., 2013). However, little is known about the mechanism underlying seed color formation in *B. napus*. Using three groups of *B*. *napus* plants in different genetic backgrounds, we showed that the 12 genes (*BnTT3, BnTT18, BnBAN, BnTT12, BnTT19, BnTT10, BnAHA10, BnTT2, BnTT8, BnTT16, BnTTG2*, and *BnTT1*) investigated in this study were highly expressed and showed clear divergence in organ specificity in the developing seed (Figure 6), suggesting that these genes play an important role in seed development and are involved in the accumulation of seed pigmentation. Furthermore, *BnTT15* was highly expressed in flowers (Figure 6R). By contrast, *BnTT5, BnTT7*, and *BnTTG1* expression did not differ significantly (Student's *t*-test, *P* > 0.05) among *B*. *napus* organs (Figures 6B,D,N). Similar expression profiles were observed for orthologs of these genes in *A. thaliana* (Schmid et al., 2005), suggesting evolutionary conservation of the regulatory mechanism governing flavonoid accumulation. Our study lays the foundation for future research aimed at deciphering the expression profiles of different gene copies in *B. napus*. These findings provide insight into the characteristics and functions of flavonoid pathway genes in *B*. *napus*.

**Figure 8:**
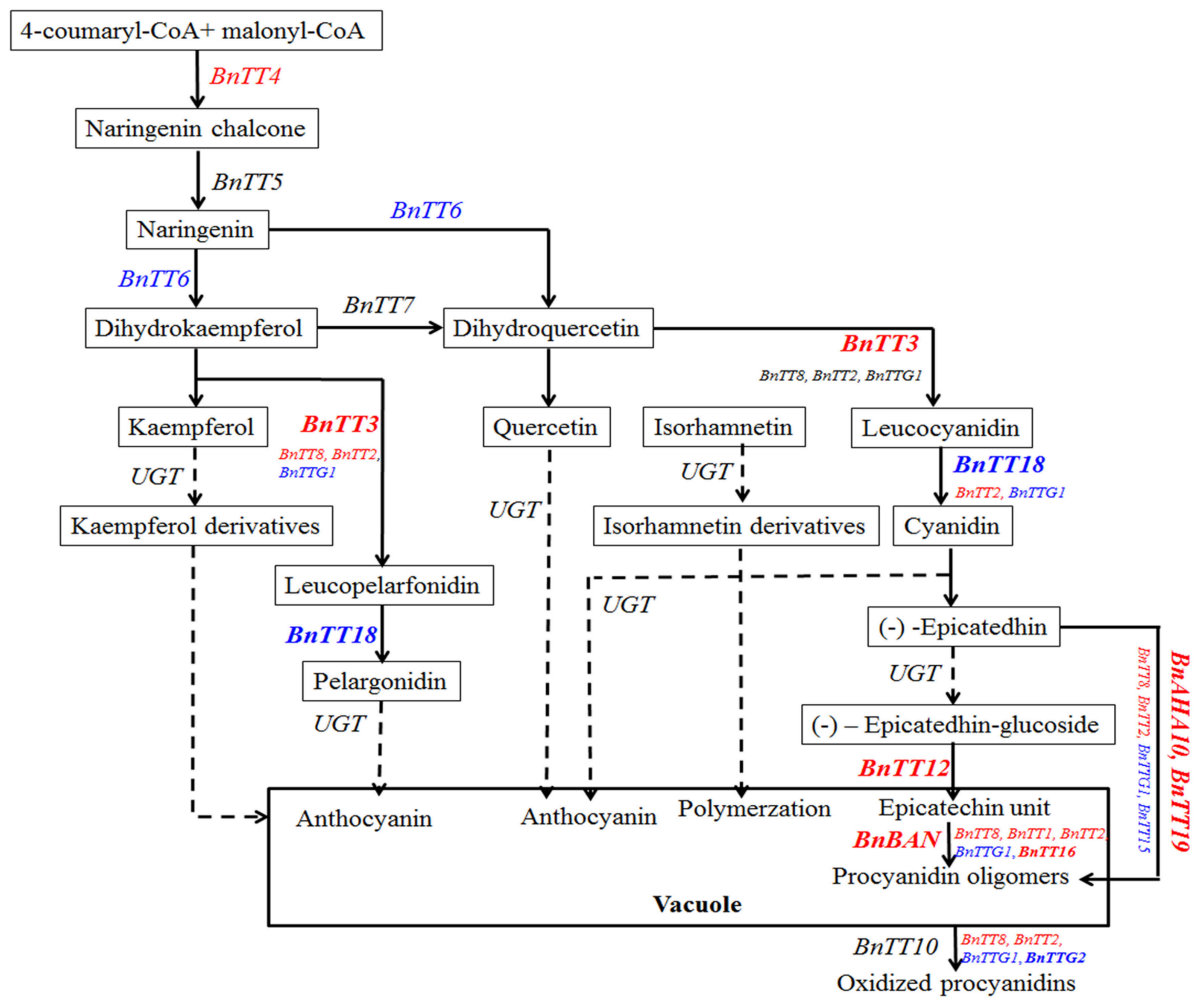
**The pathway of flavonoid biosynthesis, modification, and transport in *B. napus***. *Bn, Brassca napus*; *BAN, BANYULS*; *AHA10*, Autoinhibited H^+^-ATPase isoform 10; *TT*(*G*), *TRANSPARENT TESTA* (*GLABRA*); UGT, UDP flavonoid glucosyl transferase. The the Early Biosynthetic Genes (EBGs) and the Late Biosynthetic Genes (LBGs) red and blue colors, respectively. The bold means that genes might play an crucial roles in the formation of different seed coat colors in *B. napus.*

As in *A. thaliana*, the flavonoid biosynthesis pathway has been characterized mainly using *tt* mutants that exhibited a transparent and colorless testa (seed coat; Yu, 2013). Moreover, much research has focused on identifying the seed pigments involved in the formation of seed coat color in *B*. *napus* (Theander et al., 1977; Marles and Gruber, 2004; Akhov et al., 2009; Qu et al., 2013). Homologous genes in the *B*. *napus* flavonoid biosynthesis pathway have also been cloned and characterized (Wei et al., 2007; Xu et al., 2007; Ni et al., 2008; Akhov et al., 2009; Auger et al., 2009; Chai et al., 2009; Lu et al., 2009; Chen et al., 2013). However, a few of these genes was comprehensively functionalized in *B. napus*. Using qRT-PCR analysis, we now examined the temporal and spatial expression patterns of 18 flavonoid biosynthesis genes in the developing seeds of black- and yellow-seeded *B. napus*, sourced from different backgrounds. We found that the majority of genes had similar expression patterns in the developing seed, suggesting that these genes not only participated in the flavonoid pathway, but also might be regulated by an upstream regulatory gene involved in seed coat color formation in *B*. *napus*. In addition, we classified these genes based on the time at which their expression peaked. The first group of genes with similar expression patterns in black- and yellow-seeded lines (*BnTT4, BnBAN, BnTT12, BnTT19, BnAHA10, BnTT8, BnTT16, BnTTG2*, and *BnTT15*) was expressed at different levels in different tissues and showed the highest expression levels at 20 DAP, and was considered as the early biosynthetic genes (Figures 7, 8). By contrast, *BnTT4, BnTT12*, and *BnAHA10* have higher expression levels in black-seeded than in yellow-seeded lines (Figures 7A,H,K). The first dedicated step for flavonoid biosynthesis of plant is catalyzed by *TT4*, which produces naringenin chalcone, and thus *TT4* critically influences many important flavonoid-related characteristics, such as seed coat color, flower color, and pigmentation of the stem and leaf surface (Hoffmann et al., 2006; Kasai et al., 2007). Chai et al. (2009) proposed *BnTT12* a potential candidate gene for seed coat color formation in *B*. *napus*. Stein et al. (2013) found that the transparent testa gene *AHA10* strongly affected both seed color and lignin content using a marker that was closely linked to a major QTL for seed fiber and color in *B*. *napus*. Moreover, *TT12* and *AHA10* in *Arabidopsis* were both found to be related to the vacuolar transport of proanthocyanidin in seed coats (Debeaujon et al., 2001; Baxter et al., 2005). Thus, the difference in seed coat color between black- and yellow-seeded rapes seems to be related to the reduction in precursor accumulation following down-regulation of the encoding gene (Figure 8).

Another group of genes (*BnTT5, BnTT7, BnTT18, BnBAN, BnTT19, BnTT2*, and *BnTTG2*) exhibited drastic differences during the middle to late stages of seed coat development between the black- and yellow-seeded *B*. *napus* (Figures 7B,D,F,G,I,L,P). Additionally, *TTG1, TT2*, and *TT8* were found to modulate the activity of proteins encoding *TT3* (DFR), *TT18* (LDOX), *BAN*, and *TT12* in the proanthocyanidin subpathway, respectively. Moreover, we previously found that PAs and polyphenol compounds gradually increase during seed maturation and result in significant differences (Student's *t*-test, *P* < 0.05 or 0.01) in the colors of black- and yellow-seeded *B*. *napus* seed coats (Qu et al., 2013). However, *BnTT5* and *BnTTG1* were found to possess different expression patterns among the rapeseed seeds, suggesting that they were largely influenced by the genetic background (Figures 7B,N). These data suggest that numerous compounds accumulate in the seed coat during later development stage and confer color to the mature seed. Therefore, inhibiting the expression of genes involved in their biosynthesis can lead to lighter color seeds during the middle and late development stages in *B*. *napus*, and improve the nutritional quality of rapeseed oil and meal. Moreover, this can also explain why the expression of upstream genes peaked sooner than or simultaneously with the downstream genes in the developing seeds (Figures 7, 8), indicating that the upstream genes not only control the downstream genes, but also that the upstream genes cooperate in the flavonoid biosynthesis pathway. Together, the flavonoid biosynthetic pathway of *Brassica* species is much more complex than that in *A. thaliana*, with the former not only having more synthesis-related genes, but also exhibiting interactions with other genes involved in flavonoid biosynthesis at multiple loci (Figure 8). These findings provide insight into the molecular and biochemical mechanism of seed coat color development in *B*. *napus*.

## Author contributions

CQ and FF designed and wrote the manuscript. HZ and ZW performed the data mining and gene expression analysis. YZ and XW collected the flavonoid gene sequences and bioinformatics. XX and ZT carried out reagents and the field experiments. KZ and RW analyzed the accuracy data and edited Figures. KL and JL contributed to interpretation and modification of the data and manuscript. All authors read and approved the final manuscript.

### Conflict of interest statement

The authors declare that the research was conducted in the absence of any commercial or financial relationships that could be construed as a potential conflict of interest.

## Abbreviations

*TT*: *Transparent Testa*
*Bn*: *Brassica napus* L.
*TTG*: *TRANSPARENT TEST AGLABRA*
PAL: Phenylalanine ammonia-lyase
C4H: cinnamate 4-hydroxylase
FLS: flavonol synthase
LDOX: leucoanthocyanidin dioxygenase
BAN: BANYULS
AHA10: H^+^-ATPase isoform 10
PAs: proanthocyanidins
*Bj*: *Brassica juncea*
BLAST: basic local alignment search tool
BRAD: *Brassica* Database
PGDD: PLANT GENOME DUPLICATION DATABASE
DAP: days after pollination.
