# Supplementary material for: Genome-Wide Survey of Flavonoid Biosynthesis Genes and Gene Expression Analysis between Black- and Yellow-Seeded Brassica napus
Source: Front Plant Sci. 2016 Dec 6;7:1755. doi: 10.3389/fpls.2016.01755 (PMC5139615; doi:10.3389/fpls.2016.01755)
Supplement: Supplementary file 8 [file Image3.PDF]

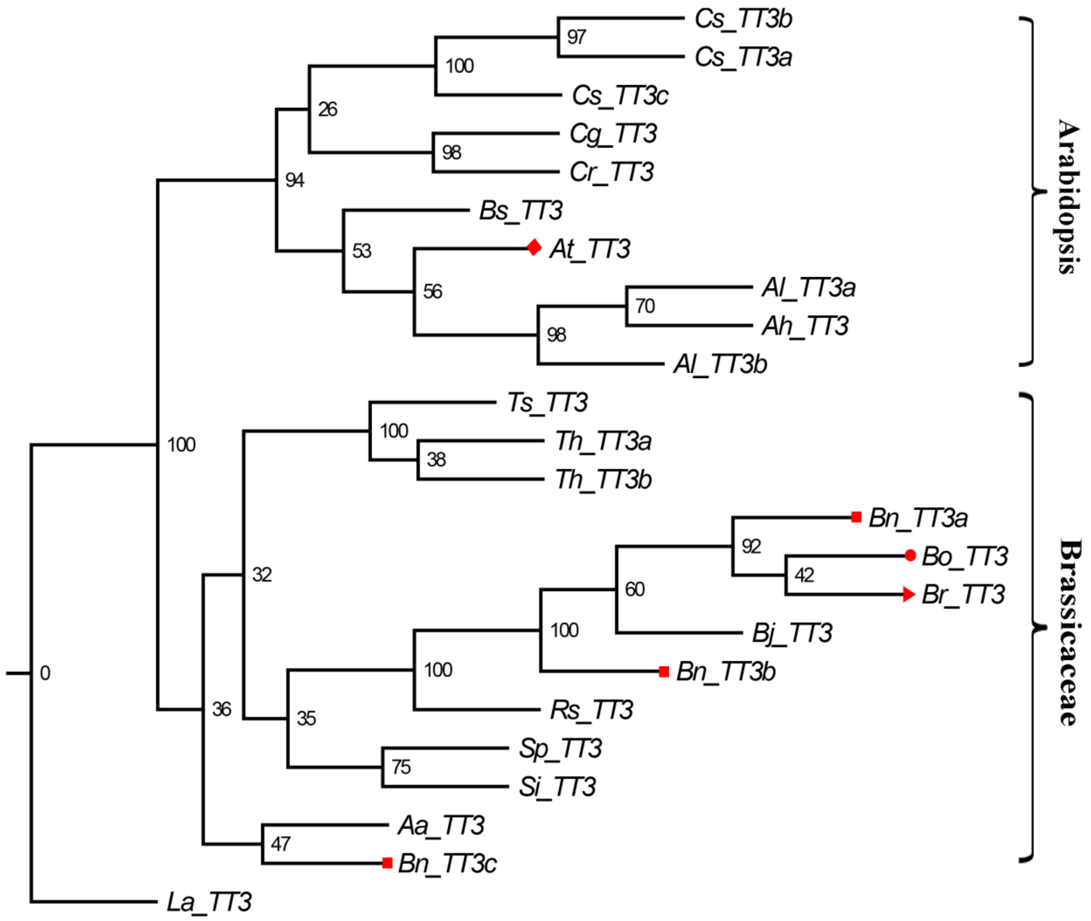

Phylogenetic relationships of *TT3* gene family of Brassicaceae species

Supplementary Figure S3 Phylogenetic relationships of *TT3* gene family of Brassicaceae species. The Red color diamond, triangle, circle, and rectangle were denoted the gene copies in *A. thaliana*, *B. rapa*, *B. oleracea*, and *B. napus*, respectively. Scale bar (the numbers) indicates the estimated number of amino acid substitutions per site.
